# Antifungal Activity and Biochemical Response of Cuminic Acid against *Phytophthora capsici* Leonian

**DOI:** 10.3390/molecules21060756

**Published:** 2016-06-11

**Authors:** Yong Wang, Yang Sun, Ying Zhang, Xing Zhang, Juntao Feng

**Affiliations:** Research and Development Center of Biorational Pesticides, Northwest A & F University, Yangling 712100, China; wy2010102163@163.com (Y.W.); SUNYANG136592@nwsuaf.edu.cn (Y.S.); zhangying19900415@126.com (Y.Z.); zhxing1952@126.com (X.Z.)

**Keywords:** antifungal activity, cuminic acid, *Phytophthora capsici*, biochemical response, defense capacity

## Abstract

Phytophthora blight of pepper caused by *Phytophthora capsici* Leonian is a destructive disease throughout the world. Cuminic acid, extracted from the seed of *Cuminum cyminum* L., belongs to the benzoic acid chemical class. In this study, the sensitivity and biochemical response of *P. capsici* to cuminic acid was determined. The mean EC_50_ (50% effective concentration) values for cuminic acid in inhibiting mycelial growth and zoospore germination of the 54 studied *P. capsici* isolates were 14.54 ± 5.23 μg/mL and 6.97 ± 2.82 μg/mL, respectively. After treatment with cuminic acid, mycelial morphology, sporangium formation and mycelial respiration were significantly influenced; cell membrane permeability and DNA content increased markedly, but pyruvic acid content, adenosine triphosphate (ATP) content, and ATPase activity decreased compared with the untreated control. In pot experiments, cuminic acid exhibited both protective and curative activity. Importantly, POD and PAL activity of the pepper leaves increased after being treated with cuminic acid. These indicated that cuminic acid not only showed antifungal activity, but also could improve the defense capacity of the plants. All the results suggested that cuminic acid exhibits the potential to be developed as a new phytochemical fungicide, and this information increases our understanding of the mechanism of action of cuminic acid against *Phytophthora capsici*.

## 1. Introduction

Pepper (*Capsicum annum* L.), one of the most important market vegetables, is grown worldwide. At present, the pepper industry of China occupies over 1.3 million hectares, producing an annual output valued at nearly 27 billion dollars [1]. However, Phytophthora blight caused by the heterothallic oomycete pathogen *Phytophthora capsici* Leonian is a major problem affecting the yield and quality of marketable peppers throughout the world [2,3,4,5,6,7,8,9]. The disease cycle is very short. In the early stages, the first signs of disease are brown necrotic areas on the root and crown of plants, after which the disease disperses rapidly to the whole plant through splashing rain or overhead irrigation waters, especially under the condition of wet soils over 18 °C and prolonged wet periods with air temperatures ranging from 24 to 29 °C [10,11,12]. The pathogen is soilborne and can infect roots, crowns and even foliar parts of pepper plants at different developmental phases [13]. It can also survive for several years as oospores in soil or as mycelium in plant residues, which are the primary forms of inocula [14].

Chemical measures remain the principal methods for control of Phytophthora blight due to limited resistant cultivars developed. The phenylamide fungicides metalaxyl or mefenoxam were the most widely used fungicides in controlling this disease [4,15], and consequently, high levels of resistance to phenylamide fungicides have been reported due to the repeated applications [16,17]. The strobilurin fungicides have also been used for control of Phytophthora blight for many years. However, the European and Mediterranean Plant Protection Organization (EPPO) has shown that *P. capsici* can easily develop resistance to this fungicide [15,18]. Moreover, isolates resistant to strobilurin fungicides have been reported in several important fungi, such as *Alternaria alternata*, *Pseudoperonospora* *cubensis*, and *Blumeria graminis* f.sp. *tritici* [19,20,21,22]. Owing to advantages in environment and health safety, and to minimize the risk of resistance development, many antifungal agents from plant extracts have been studied, such as limonoids from *Khaya ivorensis* A Chev [23], 1,2-Dihydro-6α-acetoxyazadirone from the fruit of *Chisochton paniculatus* (Roxb.) Hiern [24], and allicin from *Allium sativum* L. [25].

**Figure 1:**
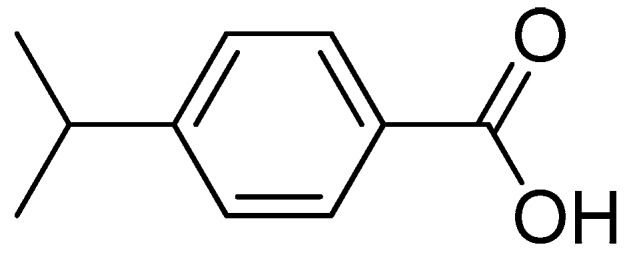
Chemical structure of cuminic acid.

Cuminic acid (*p*-isopropylbenzoic acid, Figure 1), was extracted from the seed of *Cuminum cyminum* L. [26]. Previous studies have shown that it exhibited potential antifungal activity on several plant pathogens both *in vivo* and *in vitro*, such as *Sclerotinia sclerotiorum* (Lib.) de Bary, *Gaeumannomyces graminis* var *tritici*, and *Rhizoctonia cerealis* van der Hoeven [27,28]. The mycelia growth of *S. sclerotiorum*, *R. cerealis*, and *P. capsici* were completely inhibited when treated with cuminic acid at 200 μg/mL. In greenhouse experiments, over 50% protective efficacy against *Blumeria graminis* was obtained when cuminic acid was applied at 1000 μg/mL; 57% efficacy against *S. sclerotiorum* was obtained when treated with cuminic acid at 100 μg/mL, which was equal to the efficacy obtained with procymidone at the same concentration. Moreover, the EC_50_ values of cuminic acid against *S. sclerotiorum* and *P. capsici* for mycelial growth were 7.3 and 19.6 μg/mL, respectively, which were lower than the previously reported EC_50_ values of the natural compound eugenol [28,29].

Benzoic acid is frequently used as food preservative [30]. However, cuminic acid, despite being a benzoic acid derivative, has not been registered as food preservative in China. Although previous studies had shown that it exhibited potential antifungal activity, few literature references have reported the biochemical effects or the mechanism of action of cuminic acid against phytopathogens. Therefore, the present work aimed to: (i) determine the sensitivity of *P. capsici* mycelial growth and zoospore germination to cuminic acid; (ii) evaluate the effect of cuminic acid on the morphological and physiological characteristics of *P. capsici*. This information will provide new reference data for further investigation of the mode of action of cuminic acid against *P. capsici* and other phytopathogens.

## 2. Results

### 2.1. Sensitivity to Cuminic Acid and Metalaxyl

**Figure 2:**
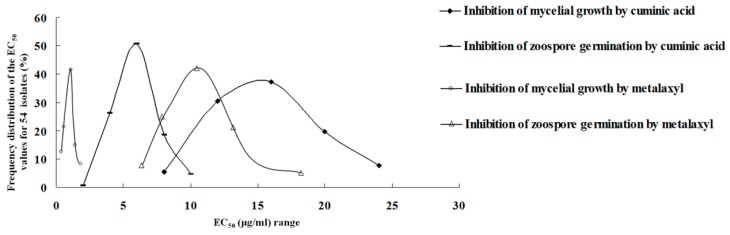
Frequency distributions of EC_50_ values of 54 *P. capsici* isolates for cuminic acid and metalaxyl for inhibition of mycelial growth and zoospore germination, respectively.

The EC_50_ values of cuminic acid for inhibition of mycelial growth of 54 *P. capsici* isolates ranged from 6.87 to 23.75 μg/mL, and the average EC_50_ value was 14.54 ± 5.23 μg/mL. Furthermore, EC_50_ values of cuminic acid for inhibition of zoospore germination of the 54 *P. capsici* isolates examined ranged from 2.86 to 9.64 μg/mL and the average EC_50_ value was 6.97 ± 2.82 μg/mL, indicating that cuminic acid had a strong inhibitory effect on *P. capsici* zoospore germination (Figure 2).

The EC_50_ values of metalaxyl for inhibition of mycelial growth and zoospore germination ranged from 0.089 to 1.71 μg/mL and 5.17 to 18.75 μg/mL with average EC_50_ values of 0.144 ± 0.053 μg/mL and 11.72 ± 3.62 μg/mL, respectively. All the distributions of the EC_50_ values were unimodal (Figure 2).

### 2.2. Effect of Cuminic Acid on Mycelial Morphology and Sporangium Formation

**Figure 3:**
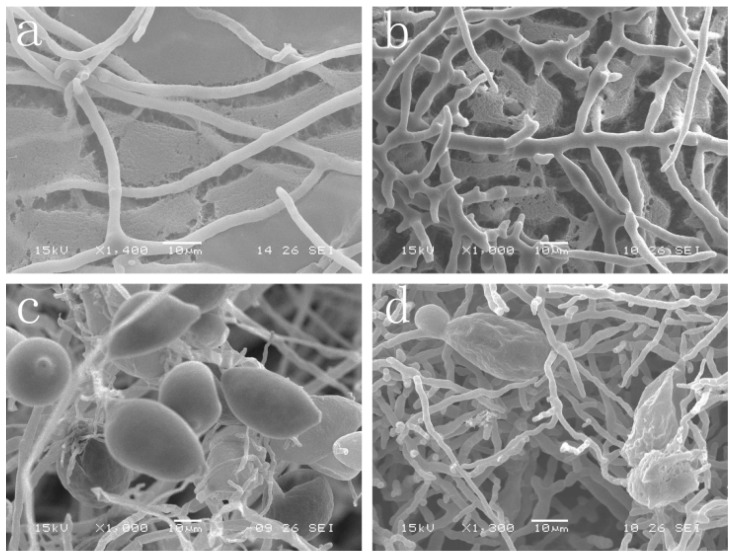
Effect of cuminic acid on (**a**,**b**) mycelial morphology and (**c**,**d**) sporangium formation of *P. capsici*. (**a**,**c**): Untreated plates; (**b**,**d**): Plates treated with cuminic acid at 20 μg/mL.

The ultrastructure of *P. capsici* mycelia treated with cuminic acid was observed by SEM. After treatment with cuminic acid, mycelia were severely deformed, displaying excessive branching. Moreover, the surface of mycelia had numerous protuberances and intensive hyphal tops, while untreated plates appeared natural (Figure 3a,b). In addition, formation of sporangia by *P. capsici* was natural when plates were not treated with cuminic acid; whereas, sporangium formation by *P. capsici* was significantly influenced by cuminic acid at 20 μg/mL (Figure 3c,d).

### 2.3. Mycelial Respiration

**Figure 4:**
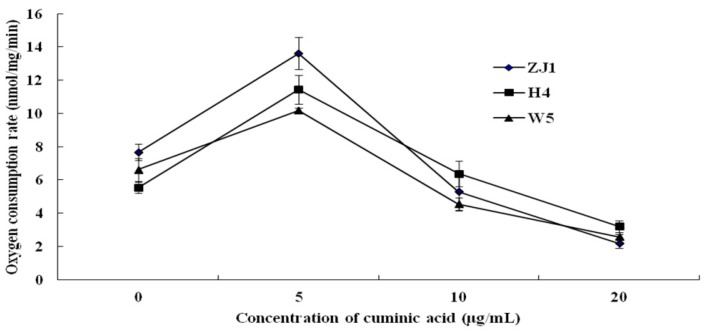
Effect of different concentrations of cuminic acid on mycelial respiration of three *P. capsici* isolates ZJ1, H4, and W5. Values are means and standard errors.

Mycelial respiration tests of *P. capsici* showed that the oxygen consumption rate was disparate when treated with cuminic acid at different concentration. When treated with cuminic acid at 5 μg/mL, the oxygen consumption rate of mycelia was higher than that of untreated control. Interestingly, when treated with cuminic acid at 10 μg/mL, the oxygen consumption rate of mycelia decreased. Mycelial respiration was significantly suppressed when treated with cuminic acid at 20 μg/mL (Figure 4). ZJ1 and H4 were randomly selected isolates, W5 was the most insensitive to metalaxyl among the 54 *P. capsici* isolates. However, the trends of respiration rate were uniform. Together, these data suggested that low concentrations of cuminic acid might enhance the mycelial respiration, while mycelial respiration could be inhibited when treated with cuminic acid at critical concentrations.

### 2.4. Cell Membrane Permeability

**Figure 5:**
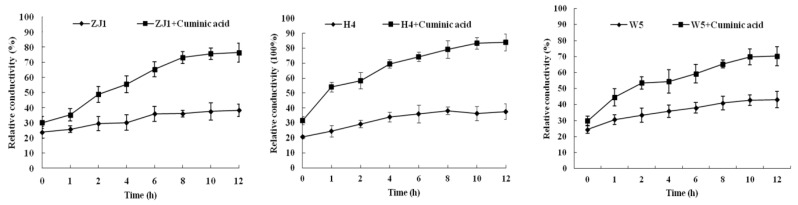
Mycelial relative conductivity of three wide-type *P. capsici* isolates ZJ1, H4, and W5 with or without cuminic acid treatment. Values are means and standard errors.

As shown in Figure 5, the relative conductivity of *P. capsici* mycelia increased over time whether treated with cuminic acid or not. After treatment with cuminic acid, the cell membrane permeability of *P. capsici* isolates ZJ1, H4, W5 was always higher than that of the untreated controls. After 10 h, the relative conductivity was stable. These results suggested that cuminic acid might lead to cell membrane damage and mycelia electrolyte leakage increase of *P. capsici*.

### 2.5. DNA and Pyruvic Acid Content

**Figure 6:**
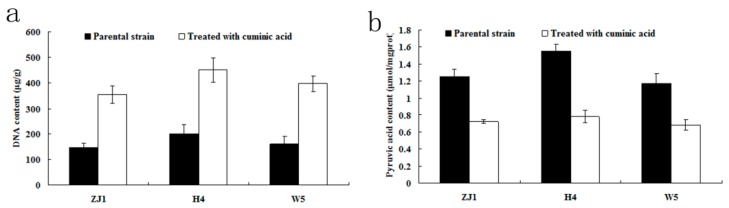

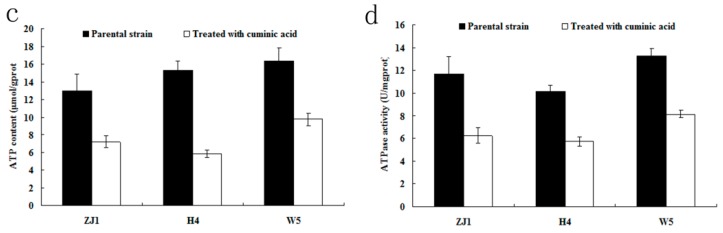
Comparison in DNA content, pyruvic acid content, ATP content, and ATPase activity of mycelia of three *P. capsici* isolates ZJ1, H4, and W5 treated with or without cuminic acid. (**a**) DNA content; (**b**) pyruvic acid content; (**c**) ATP content; and (**d**) ATPase activity. Values are means and standard errors.

When treated with cuminic acid, the DNA contents of the three isolates ZJ1, H4, W5 were significantly higher than that of the control (Figure 6a). In contrast with DNA content, pyruvic acid contents of the three isolates ZJ1, H4, W5 treated with cuminic acid were different from the control, and were reduced by 42.4%, 49.68%, and 41.88%, respectively (Figure 6b).

These data indicated that cuminic acid might inhibit the synthesis of pyruvic acid and affect the metabolism of DNA in *P. capsici*.

### 2.6. ATP Content and ATPase Activity

ATP is the direct source and a classical indicator of energy needed by all life organisms *in vivo*. Moreover, ATPase activity is a critical indictor of the rate of energy utilization [31]. After treatment with cuminic acid, ATP contents were significantly lower than that of the control (Figure 6c). As showed in Figure 6d, ATPase activities of the three isolates ZJ1, H4, W5 treatment with cuminic acid were significantly lower than that of the control. These data suggested that cuminic acid could not only decrease the activity of ATPase, but also inhibit the energy production in *P. capsici*.

### 2.7. Protective and Curative Activity of Cuminic Acid

**Figure 7:**
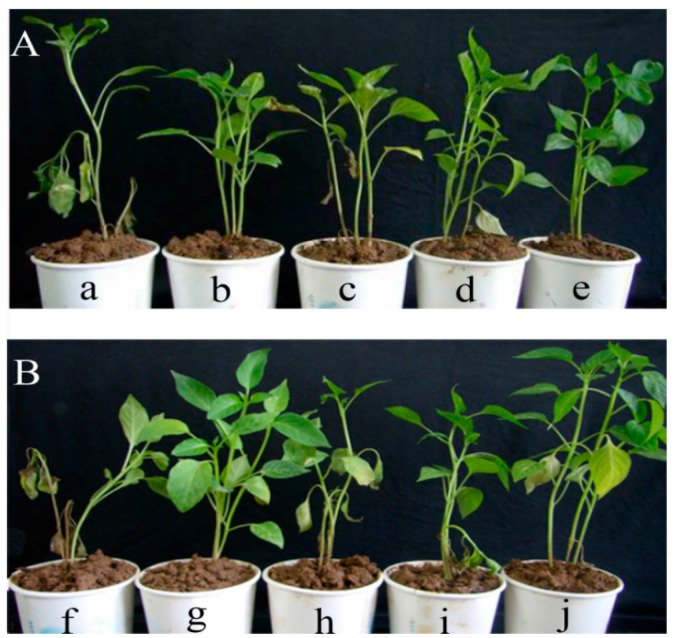
Protective (**A**) and curative (**B**) activity of cuminic acid. a, f: water control; b, g: treated with metalaxyl at 250 μg/mL; c, h: treated with cuminic acid at 250 μg/mL; d, i: treated with cuminic acid at 500 μg/mL; e, j: treated with cuminic acid at 1000 μg/mL.

The pepper seedlings died whether irrigated with spore suspension before or after irrigating with water. With an increased concentration of cuminic acid, the plants grew better and better (Figure 7). When irrigated with cuminic acid at 1000 μg/mL, over 65% protective and curative efficacies were obtained. Importantly, there was no significant difference between the protective activity of cuminic acid at 1000 μg/mL and metalaxyl at 250 μg/mL. In addition, the efficacy obtained by cuminic acid for protective activity was always higher than that for curative activity at the same concentration (Table 1).

### 2.8. Peroxidase (POD) and Phenylalanine Ammonia-Lyase (PAL) Activity

**Figure 8:**
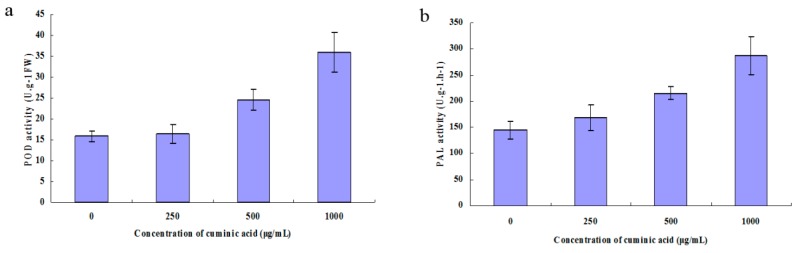
POD (**a**) and PAL (**b**) activity of the pepper leaves treated with cuminic acid. Values are means and standard errors.

There was no significant change in the activity of POD and PAL when treated with cuminic acid at low concentration. With the increased concentration of cuminic acid, POD and PAL activities increased markedly (Figure 8).

## 3. Discussion

The heterothallic oomycete pathogen *P. capsici* that is the causative agent of Phytophthora blight has developed resistance to phenylamide fungicides worldwide and control failures have occurred in the field [17]. Cuminic acid, belonging to the benzoic acid chemical class, was extracted from the seed of *Cuminum cyminum* L [26]. Previous studies had demonstrated that cuminic acid exhibited broad-spectrum antifungal activity and had better protective than curative activity against *B. graminis* [28]. In this study, we determined the effect of cuminic acid against *P. capsici* for inhibition of mycelial growth and zoospore germination. Although the 54 *P. capsici* isolates were collected from different places in Jiangsu Province, the distribution of the EC_50_ values for cuminic acid was unimodal over a sensitive range, indicating no resistant subpopulations among the isolates used in this study.

Numerous studies have demonstrated that sporangium formation and zoospore release are critical in all stages in the life cycle of *P. capsici*, which could provide the greatest opportunity for a rapid accumulation in the number of infective propagules and subsequent higher potential for host infection, disease occurrence and prevalence [17,32]. Therefore, introduction of fungicides that can disrupt the life cycle of *P. capsici* is recommended for consistent control of Phytophthora blight of pepper. As expected, in the present study, the mean EC_50_ values for cuminic acid in inhibiting zoospore germination were lower than that for inhibiting mycelial growth, indicating that zoospores were more sensitive to cuminic acid than mycelia. Although a previous study had indicated that the natural compound eugenol also exhibited antifungal activity against *P. capsici,* it had no activity on inhibition of zoospore germination [29]. Interestingly, the average EC_50_ value for cuminic acid in inhibiting mycelial growth of *P. capsici* was higher than that for metalaxyl, while the average EC_50_ value for cuminic acid in inhibiting zoospore germination was significantly lower than that for metalaxyl. These data suggested that if cuminic acid were applied in field, the life cycle of *P. capsici* might be disrupted due to the inhibition of zoospore germination, and that cuminic acid might have novel mode of action which was different from those of metalaxyl or eugenol. In addition, the efficacies for protective activity by cuminic acid were always higher than that for curative activity at the corresponding concentration. All the above indicated that cuminic acid has potential as a protective fungicide.

Benzoic acid has been commonly used as food preservative [30]. It inhibits the absorption of amino acids by interfering with the cell membrane permeability. Moreover, the enzyme activity of respiration and condensation reaction of acetyl coenzyme A could also be inhibited by benzoic acid at an intracellular level [30,33]. In the present work, after treatment with cuminic acid, mycelial morphology and sporangium formation were seriously affected, which might lead to damage of the mycelial structure. Then the increased mycelia electrolyte leakage might lead to enhanced relative conductivity of mycelia. In addition, DNA content increased markedly when treated with cuminic acid, indicating that DNA metabolism of *P. capsici* might be disordered and then accumulate. Pyruvic acid is the final product of the glycolytic pathway in glucose metabolism along with the generation of ATP [34]. Under aerobic conditions, pyruvic acid could be oxidized to produce acetyl CoA which is involved in the tricarboxylic acid cycle [35,36]. Decreased ATP content and ATPase activity after treated with cuminic acid was correlated with the decreased content of pyruvic acid. In addition, a similar phenomenon compared was observed with benzoic acid when the mycelia were treated with cuminic acid. These results suggested that the action mechanism of cuminic acid was similar with benzoic acid.

POD and PAL play important roles in plant defense. POD can enhance the woody content of young plant tissue, and oxidize phenols to quinones [37]. PAL is correlated with the plant resistance stress reaction in phenylpropanoid metabolism and plays an important role in the formation of secondary substances [38]. POD and PAL activities were increased after treated with cuminic acid, which indicated that cuminic acid could enhance the defense capacity of plants.

At present, there are nearly five hundred thousand plants throughout the world and over 1300 secondary compounds from plants have antifungal activity [39]. Considering the pesticide resistance and harmfulness to the environment, natural compounds extracted from plants are becoming more popular due to their specific antifungal activity, easy degradation, and human safety [40,41,42,43]. Moreover, natural compounds are often considered to be lead compounds for the synthesis of novel fungicides. For example, the strobilurin fungicides (azoxystrobin, picoxystrobin) were synthesized from strobilurin A [44]; fenpiclonil was synthesized from pyrrolnitrin [45]. Considering the potential antifungal activity, synthesis of new fungicides based on the structure of cuminic acid is underway in our laboratory.

In summary, all the data suggest that cuminic acid extracted from the seed of *Cuminum cyminum* L. exhibited the antifungal activity and show the potential value as a natural alternative to commercial fungicides for the control of Phytophthora blight of pepper. Importantly, cuminic acid can enhance the defense capacity of plants. Evaluation of cuminic acid for the control of some plant diseases caused by *P. capsici*, *S. sclerotiorum* are in progress in field experiments. The biochemical influence on *P. capsici* by cuminic acid suggests that it might inhibit energy generation and target genes correlated with the glycolytic pathway. Further study is still needed. These results should be valuable for fully understanding the mode of action of cuminic acid and useful for the development of new antifungal drugs.

## 4. Materials and Methods

### 4.1. Media, Pathogen and Fungicides

V-8 juice medium was prepared with 200 mL of V-8 juice, 2 g of CaCO_3_, 16 g of agar and 800 mL of distilled water [46]. Potato dextrose agar (PDA) was prepared with 200 g of potato, 20 g of agar, and 20 g of dextrose per liter of distilled water [47]. V-8 media were used for sporangium formation, zoospore germination and PDA was used for *P. capsici* mycelial growth.

Fifty-four single-sporangia isolates of *P. capsici*, collected from different locations in Jiangsu Province of China, were kindly provided by the Jiangsu Key Laboratory of Pesticide, College of Plant Protection, Nanjing Agricultural University and maintained on PDA slants at 4 °C. In these isolates, W5 was the most insensitive to metalaxyl.

Technical grade (98%) cuminic acid was purchased from Jianglai Biotechnology Company (Shanghai, China). Metalaxyl (98%) was provided by Wenzhou Pesticide Factory (Zhejiang, China). It was dissolved in methanol (>99.5%) to 10 mg/mL for the stock solutions, and then stored at 4 °C in the dark.

### 4.2. Determination of Sensitivity of Mycelial Growth to Cuminic Acid

Sensitivity of 54 *P. capsici* isolates to cuminic acid by mycelial growth was determined as follows: fresh mycelial plugs (5 mm in diameter) cut from the leading edge of an actively growing colony were transferred to a series of PDA plates containing 0, 3.125, 6.25, 12.5, 25 or 50 μg/mL cuminic acid. After 4 days of incubation in a growth chamber at 25 °C, colony diameter was measured by measuring the average diameter in two perpendicular directions. The EC_50_ values were calculated by regressing percentage growth inhibition against the log of fungicide concentration according to previous studies [15,47]. Each concentration has three replicates and the experiment was conducted three times.

### 4.3. Determination of Sensitivity of Zoospore Germination to Cuminic Acid

Samples of zoospores were prepared as follows: to induce sporangium formation, six mycelial plugs cut from each 1 to 2-week-old V-8 Petri dish were covered with sterile distilled water and kept in a growth chamber (12 h photoperiod) at 25 °C [15]. After 4 days, plenty of sporangia were produced. To encourage the release of zoospores, the culture with sporangia was placed at 4 °C for 30 min, and then incubated at 25 °C for 1 h. After zoospores were collected, V-8 juice broth containing 0, 3.125, 6.25, 12.5, 25, and 50 μg/mL cuminic acid were added to equal volumes of the zoospore suspensions (1 × 10^5^), giving final concentrations of 0, 1.5625, 3.125, 6.25, 12.5, and 25 μg/mL (pre-test concentration) cuminic acid. After incubation for 1 h at 25 °C in darkness, the germination of zoospore was checked under an Olympus light microscope with the appearance of mycelial growth arising from germinating zoospore cysts. Germination was quantified at three sites by counting 200 zoospores per site. Then the inhibition rate of zoospore germination was determined and the EC_50_ values were calculated [48,49]. The experiment was performed twice with three plates per treatment.

### 4.4. Determination of Sensitivity of Mycelial Growth and Zoospore Germination to Metalaxyl

In sensitivity test experiment, metalaxyl was used as the control fungicide. 0, 0.0625, 0.125, 0.25, 0.5, and 1 μg/mL were used as the pre-test concentration for inhibition of mycelial growth, and 0, 3.125, 6.25, 12.5, 25, and 50 μg/mL were used as the pre-test concentration for inhibition of zoospore germination of 54 *P. capsici* isolates. The EC_50_ values were calculated for each isolate [15].

### 4.5. Effect of Cuminic Acid on Mycelial Morphology and Sporangium Formation of P. capsici

Mycelia plugs excised from the margin of 4-day-old colony of the isolate ZJ1 (randomly selected) were placed mycelia-side down on PDA or V-8 plates containing 20 μg/mL (The EC_50_ value for inhibition of mycelial growth) of cuminic acid. Plates without cuminic acid were used as control. After 3 days and 5 days at 25 °C in the dark, the margin of the medium area (10 mm × 10 mm) was cut and placed on slide glass and the mycelial morphology and sporangium formation of *P. capsici* treated with cuminic acid was observed by scanning electron microscope (GeminiSEM 300/VP, Carl Zeiss Jena, Thuringia, Germany), respectively [15,50]. There were three replicates and the experiment was conducted twice.

### 4.6. Effect of Cuminic Acid on Mycelial Respiration

Mycelial respiration was determined using the method reported previously [51,52]. Ten mycelial plugs (5 mm diameter) excised from the margins of 4-day-old colonies of each isolate ZJ1, H4 (randomly selected), W5 (most insensitive to metalaxyl) on PDA were transferred to 250-mL flasks containing 100 mL of PDB (PDA without agar). After the flasks were shaken at 175 rpm and 25 °C for 48 h, flasks were amended with cuminic acid at ultimate concentrations of 0, 5, 10, and 20 μg/mL. After shaking for another 2 h, the oxygen consumption rate by mycelia was measured with an oxygraph system (F12-ED, Julabo, Beijing, China).

### 4.7. Effect of Cuminic Acid on Cell Membrane Permeability

Three isolates ZJ1, H4 (randomly selected), W5 (most insensitive to metalaxyl) were used in this test. For each isolate, ten mycelial plugs were transferred to 250-mL flasks containing 100 mL of PDB. After the flasks were shaken at 175 rpm and 24 °C for 3 days, partial flasks were amended with cuminic acid at the concentration of their EC_50_ values. Flasks without cuminic acid were used as control. After 24 h, mycelia were collected. Then 0.5 g of fresh mycelia per sample was suspended in 25 mL of distilled water. Conductivity of the distilled water was measured after 1, 2, 4, 6, 8, 10 and 12 h with a conductivity meter (CON510 Eutech/Oakton, Singapore). After 12 h, the mycelia were boiled for 5 min to measure the final conductivity [36]. Three flasks for each treatment were used, and the experiment was performed three times. The relative conductivity of mycelia was calculated as follows:

Relative conductivity = Conductivity at different times/Final conductivity × 100%

### 4.8. Pyruvic Acid and DNA Content of Mycelia

Mycelia were collected as described above. Fresh mycelia samples were ground in liquid nitrogen. Then 0.5 g of mycelia powder per sample was transferred to a 1.5 mL centrifuge tube containing 1 mL Tris-HCL (50 mM, PH 7.5). After centrifuge at 1500 g for 10 min, pyruvic acid content was measured using a commercial assay kit (Jiancheng, Nanjing, China) according to the manufacturer’s instructions and absorbance of the solution was measured at 505 nm [53]. Three replicates for each treatment were used and the experiment was conducted twice.

In addition, 0.5 g of mycelia powder per sample obtained above was used for DNA extraction. Genomic DNA was extracted by a CTAB method according to previous studies [50,54]. Then the DNA samples were dissolved with 50μL of distilled water and the DNA content was determined using a spectrophotometer.

### 4.9. ATP Content and ATPase Activity

ATP content and ATPase activity in *P. capsici* mycelia were determined using a previously reported method [55]. Mycelial powders were prepared as described above. ATP content and ATPase activity were determined using commercial kits (Nanjing Jiancheng; Innova Biosciences, Shanghai, China) according to the manufacturer’s instructions, respectively. ATP content was defined as mmol per mg protein for tissue samples and the activity of ATPase was defined as 1 μmol inorganic phosphorus catalyzed by this enzyme in 1 mg protein in 1 h (μmol Pi/mg pro/h). There were three replicates for each isolate and the experiment was conducted twice.

### 4.10. Protective and Curative Activity of Cuminic Acid in Pot Experiments

The protective and curative activity of cuminic acid against *P. capsici* was tested according to a previous study with some modifications [56]. For protective activity, pepper seedlings with at least four true leaves were irrigated with 10 mL of water, metalaxyl at 250 μg/mL, cuminic acid at 250, 500, and 1000 μg/mL, respectively. After 24 h, pepper seedlings were irrigated with 10 mL of spore suspension (1 × 10^5^). For curative activity, pepper seedlings were irrigated with the treatment as above at 24 h after irrigated with spore suspension (1 × 10^5^). Then the irrigated plants were kept at 25 °C with 85% humidity for 20 day. The disease index and control efficacy were calculated [56]. Three pepper seedlings per pot and three pots per concentration were used. The experiment was repeated twice.

### 4.11. Peroxidase (POD) and Phenylalanine Ammonia-Lyase (PAL) Activity

Pepper leaves cut from the plants treated with cuminic acid in the above section were broken on ice. POD and PAL activity were determined using commercial kits (Jiancheng) according to the manufacturer’s instructions. One unit of POD activity was defined as a change of one in absorbance per min; one unit of PAL activity was defined as the increase of one in absorbance per h [37]. Five leaves per treatment were used and the experiment was repeated twice.

### 4.12. Statistical Analysis

Statistical analysis of data was performed using the Sigmastat statistical software package (SPSS 14.0, IBM, Chicago, IL, USA). EC_50_ values of the isolates were calculated by linear regression of the log of the colony diameter or zoospore germination *versus* the fungicide concentrations. When the ANOVA was significant, means were separated with the least significant difference test (LSD, *p* = 0.05).

## Figures and Tables

**Table 1:** Protective and curative activity of cuminic acid against *P. capsici.*

Treatment	Protective Activity		Curative Activity	
	Disease Index	Control Efficacy (%)	Disease Index	Control Efficacy (%)
Cuminic acid (250 μg/mL)	62.78b ^a^	32.00c	71.36b	21.97d
Cuminic acid (500 μg/mL)	49.25c	46.65b	51.54c	43.64c
Cuminic acid (1000 μg/mL)	26.87d	70.89a	30.28d	66.89b
Metalaxyl (250 μg/mL)	25.78d	72.08a	24.35e	73.37a
Water control	92.32a	-	91.45a	-

Note: ^a^ Values are means of three pepper seedlings and from two independent experiments. Values in each column followed by the same letter were not significant differences according to LSD (least significant difference) tests at *p* = 0.05.
